# Superonasal orbital traumatic neuroma mimicking a dermoid cyst in a young patient: A case report

**DOI:** 10.5339/qmj.2026.48

**Published:** 2026-06-29

**Authors:** Ahmad Fateh Khader, Abdulaziz Fahad A Samandar, Yousof Mohammad Shah

**Affiliations:** 1 Department of Ophthalmology, Security Forces Hospital, Makkah, Saudi Arabia; 2 College of Medicine, Umm AlQura University, Makkah, Saudi Arabia

**Keywords:** Traumatic neuroma, orbital diseases, dermoid cyst, orbital neoplasms, case reports

## Abstract

**Background:**

Orbital traumatic neuromas are rare lesions that can mimic common orbital pathologies, such as dermoid cysts, particularly in the superonasal region.

**Case Presentation:**

A 17-year-old female presented with a long-standing, slowly enlarging superonasal orbital mass that had initially been diagnosed as a dermoid cyst. Computed tomography revealed a well-defined, non-enhancing extraconal lesion measuring approximately 3.1×1.5×1.3 cm with associated bone remodeling. Surgical excision revealed a solid, vascular lesion complicated by retrobulbar hemorrhage, which was promptly managed. Histopathological examination confirmed a traumatic neuroma.

**Conclusion:**

Traumatic neuroma can mimic a dermoid cyst and should be considered when intraoperative findings are atypical. Definitive diagnosis relies on histopathological examination.

## 1. INTRODUCTION

Traumatic neuromas are benign, non-neoplastic, reactive proliferations of disorganized nerve fibers and Schwann cells that develop following partial or complete nerve injury, typically as a result of trauma or surgical intervention.^1^ They represent an aberrant regenerative response rather than a true neoplasm and are most commonly encountered in the extremities, head and neck region, and along major peripheral nerves.^1,2^ In the orbit, however, traumatic neuromas are exceptionally rare, with only isolated case reports described in the literature, most commonly involving the supraorbital or infraorbital nerves.^3,4^ Primary orbital neuromas account for a minute fraction of orbital tumors, making them a diagnostically obscure entity in routine ophthalmic practice.^3^

Orbital masses in the superonasal quadrant are classically associated with congenital developmental lesions rather than neural tumors. The most common diagnoses in this location include dermoid cysts, epidermoid cysts, and encephaloceles.^5,6^ Among these, dermoid cysts are the most frequent congenital orbital lesions. They result from ectodermal sequestration along embryonic suture lines and typically present as slowly enlarging, painless, firm masses in the superonasal orbit during childhood or early adulthood.^5,6^ Epidermoid cysts share a similar embryologic origin with dermoid cysts but are less common and may demonstrate a more infiltrative behavior. Encephaloceles, although rare, represent herniation of intracranial contents through bony defects and constitute an important differential diagnosis in medial and superonasal orbital masses, particularly in pediatric patients.^6^

Clinically, superonasal orbital masses often present as painless, slowly enlarging, firm lesions with minimal inflammatory signs, making differentiation based on physical examination alone challenging. On computed tomography (CT), dermoid and epidermoid cysts typically appear as well-circumscribed extraconal lesions, often associated with bony remodeling or rarefaction, and may show variable internal density depending on lipid or keratin content.^5,6^ Encephaloceles may demonstrate bony defects with continuity between intracranial and orbital compartments. In contrast, orbital neuromas lack pathognomonic imaging features and may appear as non-specific, well-defined masses, leading to frequent misdiagnosis as more common congenital lesions.^4,7^

The differential diagnosis of a superonasal orbital mass is therefore broad and includes congenital lesions (such as dermoid cysts, epidermoid cysts, and encephaloceles), inflammatory conditions, vascular lesions, neural tumors, and rare reactive processes such as traumatic neuroma.^3,5,6^ This diagnostic overlap is further compounded by the rarity of orbital neuromas and their nonspecific radiologic appearance, which contributes to delayed or incorrect diagnosis.

This report presents a rare case of a solitary superonasal orbital traumatic neuroma in a young patient that was misdiagnosed as a dermoid cyst for more than a decade. By highlighting the clinical, radiological, intraoperative, and histopathological features of this case, we aim to expand the current understanding of orbital neuromas and emphasize the importance of considering rare neural lesions in the differential diagnosis of superonasal orbital masses.

## 2. CASE PRESENTATION

In November 2025, a 17-year-old female presented to the Department of Ophthalmology at Security Forces Hospital, Makkah, Saudi Arabia, with a long-standing, painless superonasal mass involving the left eye. The lesion had been present for more than 10 years, and the patient had been followed since childhood with a presumptive diagnosis of a dermoid cyst. She denied any history of ocular trauma, orbital surgery, or facial injury. She had no history of systemic illness, no clinical features suggestive of neurofibromatosis, and no relevant family history. Over the preceding six months, the lesion had demonstrated a gradual increase in size and anterior prominence, prompting the patient to seek surgical excision for cosmetic concerns.

### 2.1. Physical examination (PE)

Examination of the left eye revealed a well-defined, firm, non-tender subcutaneous nodule located in the superonasal quadrant of the upper eyelid, just superior to the lid crease. The overlying skin was intact and showed no erythema, edema, or other signs of inflammation. There was no localized warmth or tenderness. Eyelid position and function were preserved, with no lash loss or conjunctival involvement.

**Figure 1.**
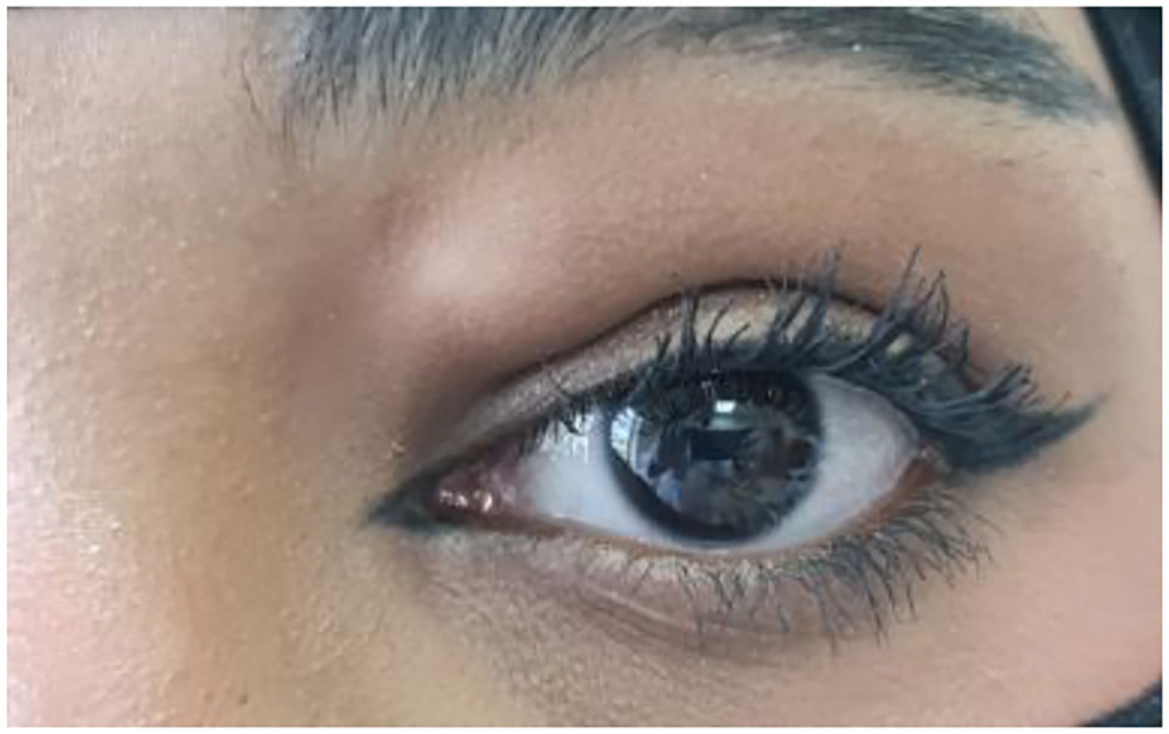
The clinical appearance of the lesion.

A complete ophthalmological examination was performed. Best-corrected visual acuity was 20/20 in both eyes. Intraocular pressure, measured by applanation tonometry, was 15 mmHg in the right eye and 16 mmHg in the left eye. Pupillary reactions were normal, with no relative afferent pupillary defect. Extraocular movements were complete in all directions of gaze, with no diplopia. Anterior segment examination was unremarkable in both eyes. Fundoscopic examination revealed normal optic discs, maculae, and retinal vasculature bilaterally. There were no signs of proptosis, globe displacement, or optic nerve compression. The clinical appearance of the lesion is shown in Figure 1.

### 2.2. Imaging

Computed tomography (CT) of the orbits demonstrated an intra-orbital, extraconal mass lesion located in the left superonasal orbit, measuring approximately 3.1×1.5×1.3 cm. The lesion showed no enhancement following intravenous contrast administration and caused mild superior displacement with slight compression of the superior rectus muscle. Associated bone rarefaction of the orbital roof was noted, extending toward the frontal sinus boundary, without evidence of intracranial extension. Both globes appeared normal, with no intraocular abnormalities. The remaining extraocular muscles and orbital fat showed normal density and morphology, and both orbital apices were clear.

**Figure 2.**
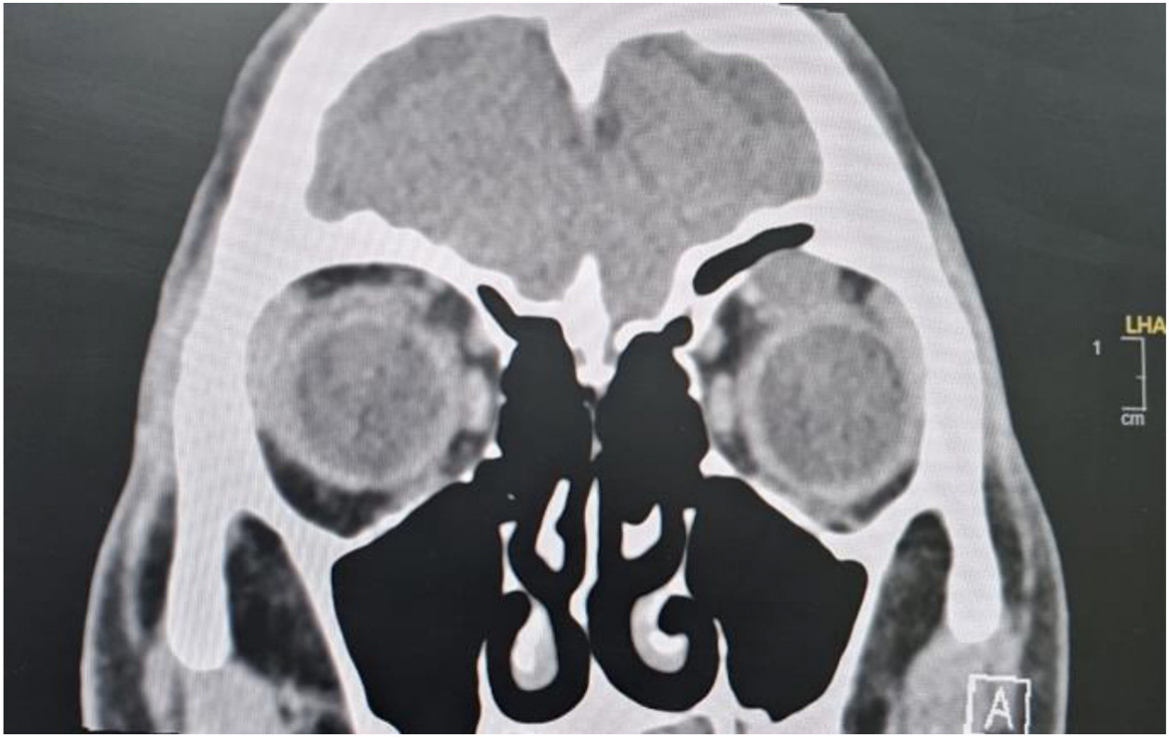
Representative CT images of the lesion.

Based on the lesion’s classic superonasal location, well-defined extraconal appearance, non-enhancing nature, and associated bony remodeling, the imaging impression was most consistent with a congenital dermoid cyst. There were no CT features suggestive of malignancy, vascular malformation, or intracranial communication. Therefore, magnetic resonance imaging (MRI) was not pursued preoperatively, as CT findings were considered sufficient for surgical planning in the context of a presumed benign congenital lesion. MRI was not expected to alter the preoperative diagnosis, surgical approach, or management strategy. Representative CT images of the lesion are shown in Figure 2.

### 2.3. Plan/management

The patient was prepared for surgical excision of the orbital mass under general anesthesia. An eyelid crease incision was selected to optimize cosmetic outcomes. Dissection was carried out through the skin and the underlying orbicularis muscle until the preseptal orbital fat was exposed, allowing access to the orbit. The mass was then identified and carefully dissected circumferentially using meticulous technique to avoid rupture of the cystic lesion.

During dissection, a vascular feeder vessel was identified at the margin of the mass—an intraoperative finding not typical of a dermoid cyst. Bleeding occurred despite the cyst remaining intact and was initially managed with cautery and suction. The mass was subsequently completely excised; however, persistent bleeding continued after removal. Additional cautery and suction were applied until hemostasis was achieved. Internal sutures were placed, followed by closure of the skin incision. The excised mass measured 2.4×1.0×0.9 cm (length×width×height). These intraoperative findings and the appearance of the excised specimen are shown inFigures 3A–C.

After surgery, the patient was transferred to the recovery room. The eye pad was noted to be blood-stained, and palpation revealed a stony-hard globe, raising concern for retrobulbar hemorrhage. Emergency lateral canthotomy and cantholysis were performed immediately, followed by reopening of the surgical wound. The wound edges were retracted, allowing evacuation of the accumulated blood from the orbit, with simultaneous external compression.

Within approximately 5 min, the globe softened, and the eyelid could be closed. The wound was subsequently re-sutured. The patient was closely monitored for signs of rebleeding, with orbital palpation performed every 15 min for the first 2 h, followed by every 30 min for the subsequent 2 h.

### 2.4. Histopathology report

**Figure 4.**
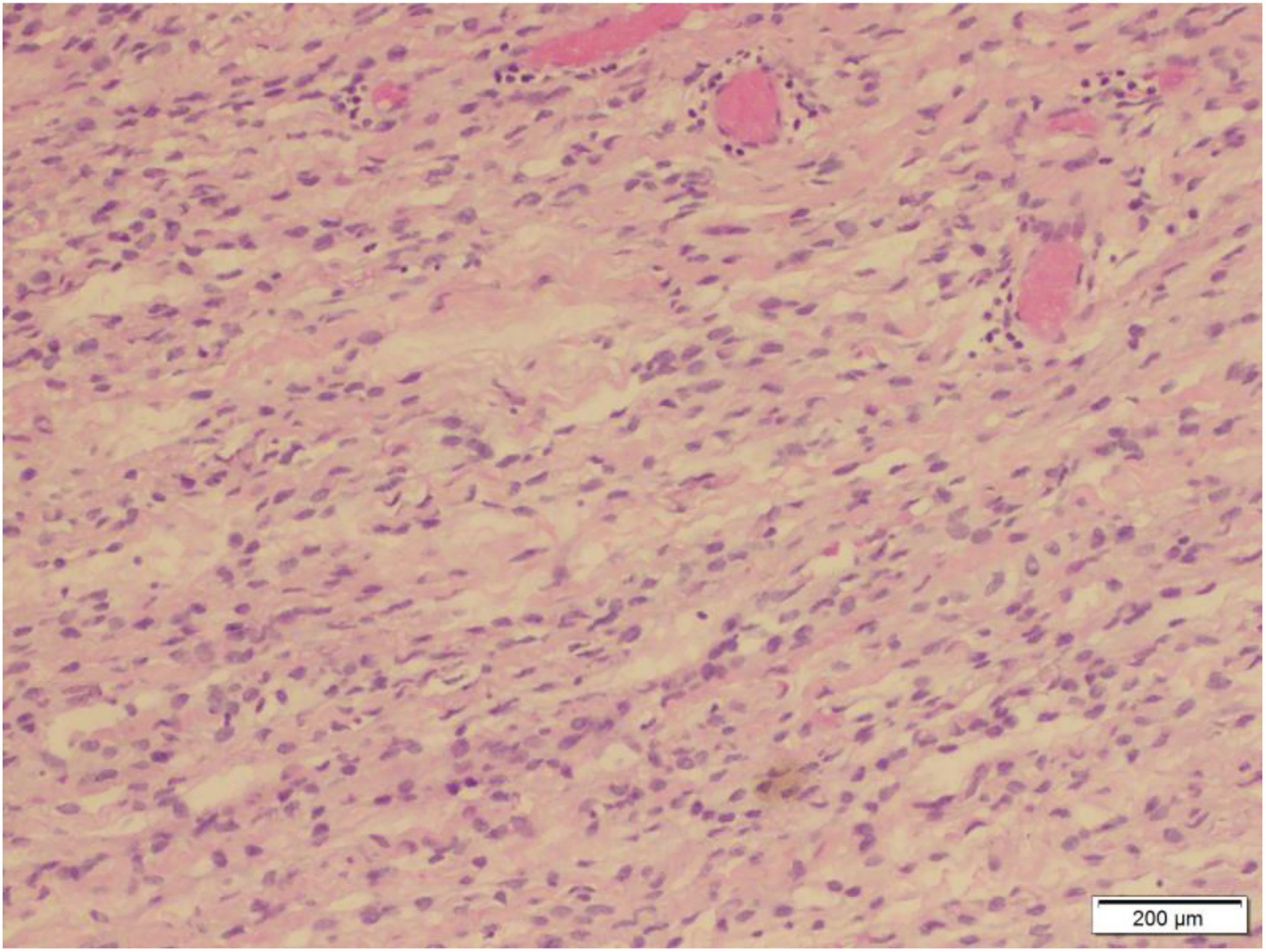
Representative microscopic features. Final Diagnosis: Traumatic neuroma.

The excised specimen was received in formalin and consisted of a well-defined, nodular gray-white mass measuring approximately 3.1 cm in greatest dimension. Microscopic examination demonstrated a lobulated, serpiginous proliferation of neural tissue composed of variable-caliber nerve bundles. These bundles were formed by benign spindle-shaped Schwann cells arranged in a haphazard microfascicular pattern, embedded within a fibrocollagenous stroma. No cytologic atypia, mitotic activity, or other features suggestive of malignancy were identified. Representative microscopic features are shown in Figure 4.

### 2.5. Postoperative first-week examination

**Figure 5.**
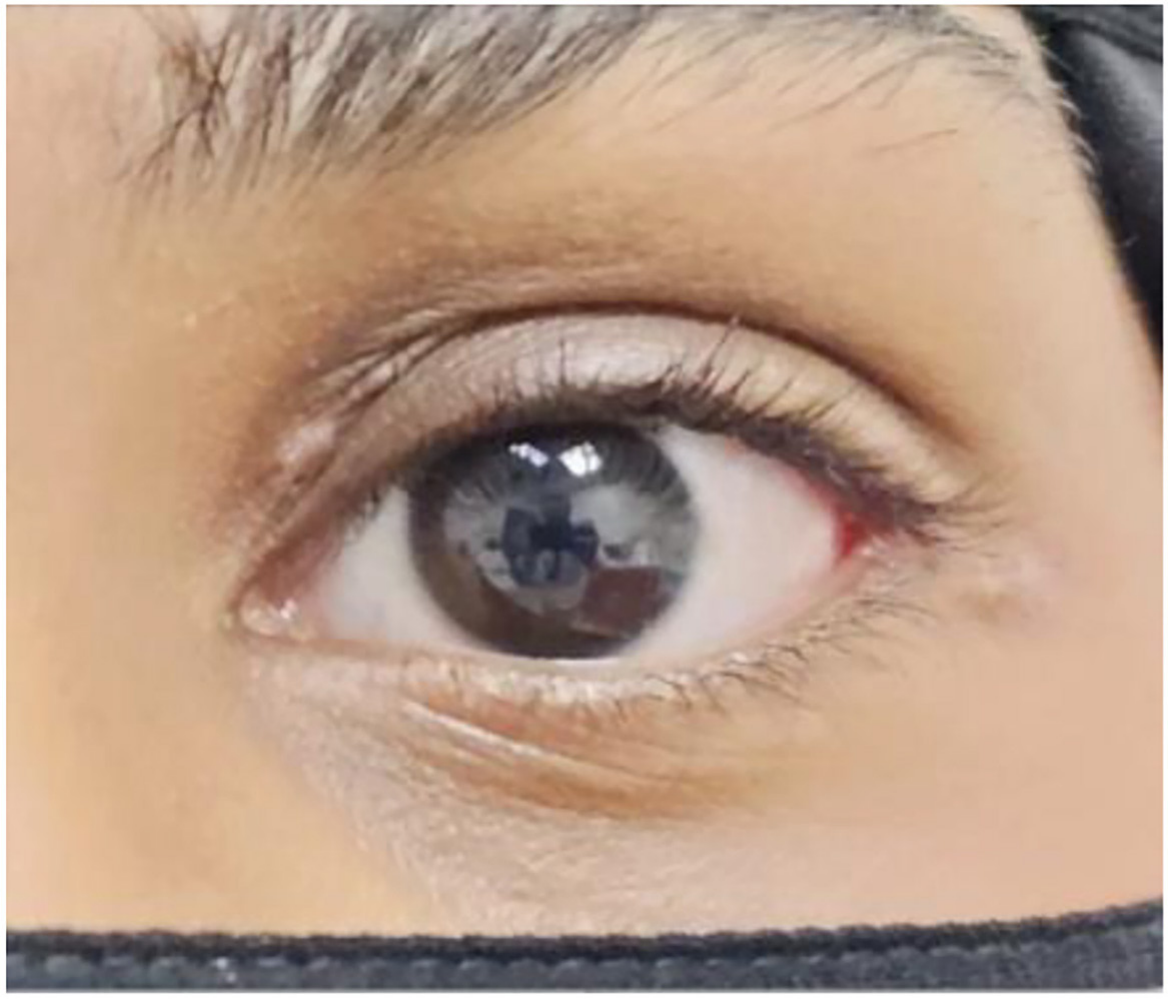
The one-week postoperative appearance. Plan: Suture removal continued ocular lubrication, dermatology consultation, evaluation to rule out neurofibromatosis, and follow-up in two weeks.

At one week postoperatively, following left superonasal intra-orbital mass excision—previously complicated by an intraoperative retrobulbar hemorrhage that was promptly and successfully managed—the patient demonstrated significant clinical improvement. Eyelid swelling and edema had resolved markedly, and the upper eyelid had almost regained its normal position, with a notable improvement in ptosis. All sutures remained intact, and the surgical wound exhibited excellent healing. The cornea showed only minimal superficial punctate staining, consistent with mild postoperative dryness. The one-week postoperative appearance is shown in Figure 5.

## 3. DISCUSSION

This report describes a rare case of a superonasal orbital traumatic neuroma in a young patient that was initially misdiagnosed as a dermoid cyst for more than a decade, underscoring important diagnostic and surgical considerations. The clinical course highlights the diagnostic challenges associated with lesions arising in anatomical locations typically associated with more common pathologies.

Traumatic neuromas are reactive proliferations of disorganized nerve tissue that usually develop following nerve injury.^1^ Their occurrence in the orbit is exceptionally rare, with most reported cases involving the supraorbital or infraorbital nerves.^1,2^ Previous reports have highlighted the rarity of orbital traumatic neuromas and their tendency to mimic other benign orbital lesions due to their non-specific clinical features.^8^ In contrast, the superonasal orbit is classically associated with congenital lesions such as dermoid cysts, which are among the most common orbital masses in this region.^5^ This anatomical predilection creates a strong diagnostic bias toward congenital etiologies and contributes to potential misdiagnosis, as demonstrated in the present case.

Compared with previously reported cases of orbital traumatic neuromas, the present case shares several important similarities and distinctions. Consistent with the existing literature, these lesions typically present as slowly enlarging, painless masses with nonspecific clinical and imaging features, often leading to misdiagnosis as benign orbital lesions.^2,3^ However, most reported cases involve the supraorbital or infraorbital nerves, whereas involvement of the superonasal quadrant is extremely uncommon.^8,9^ This unusual anatomical location, typically associated with dermoid cysts, likely contributed significantly to the prolonged misdiagnosis and highlights the influence of anatomical expectations on clinical decision-making.

On CT, the lesion appeared as a well-defined, non-enhancing extraconal mass with associated bone remodeling. These findings are non-specific and can be observed in both long-standing dermoid cysts and benign neural tumors due to chronic pressure effects.^7^ Consistent with previously reported cases, imaging alone is often insufficient to reliably distinguish traumatic neuroma from more common orbital lesions,^9^ underscoring the limited specificity of conventional imaging modalities in this context.^4,7^ Furthermore, the absence of contrast enhancement, although often considered reassuring for benign pathology, does not exclude a neural lesion, particularly in fibrotic traumatic neuromas.^2^

In retrospect, advanced imaging techniques such as MRI, including diffusion-weighted imaging and nerve-specific sequences, may provide additional diagnostic value.^7^ Previous studies have suggested that MRI can better characterize lesion composition and neural origin; however, it still lacks definitive specificity for traumatic neuroma.^9^ Therefore, the primary role of advanced imaging in such cases may be to support surgical planning and anticipate intraoperative challenges rather than to establish a definitive diagnosis.

A key distinguishing feature in this case was the unexpected intraoperative vascularity of the lesion. In contrast to dermoid cysts, which are typically cystic and avascular,^6^ the presence of a prominent feeder vessel and substantial intraoperative bleeding suggested an alternative pathology. Similar variability in vascularity has been described in neural lesions depending on the degree of fibrosis and neural proliferation.^8^ This discrepancy between the expected and the observed intraoperative findings underscores the importance of maintaining diagnostic flexibility and adapting surgical management accordingly.

The clinical implications of this finding are significant. Surgeons should remain vigilant when intraoperative features do not align with preoperative imaging, as this may indicate a different pathological entity. Preparedness for vascular complications, including early identification and control of feeding vessels, appropriate hemostatic techniques, and readiness to manage orbital compartment syndrome, is essential for ensuring patient safety.

The diagnosis was confirmed histopathologically, demonstrating characteristic features of traumatic neuroma, including haphazardly arranged nerve bundles within a fibrocollagenous stroma and no evidence of malignancy.^1^ As described in the literature, histopathological evaluation remains the gold standard for diagnosis, given the lack of specific clinical or imaging features.^1,4^ Additional reports emphasize the importance of clinicopathological correlation in establishing a definitive diagnosis.^8^

An important consideration is the exclusion of neurofibromatosis type 1 (NF1), as orbital neural lesions may be associated with this systemic condition.^4,8^ In the present case, the absence of clinical features and a relevant family history supported the diagnosis of a sporadic, isolated lesion.

The occurrence of retrobulbar hemorrhage in this case represents a significant surgical complication, likely related to the lesion’s vascularity. Similar complications have been reported during orbital surgeries involving vascular or solid lesions, highlighting the importance of early recognition and prompt intervention to prevent vision-threatening consequences.^9^

The strengths of this report include comprehensive clinical, radiological, intraoperative, and histopathological documentation. Its limitations include reliance on CT imaging without preoperative MRI. In addition, the exact etiology remains unclear, as no definite history of prior trauma was identified.^2^

In light of these findings, clinicians should maintain a high index of suspicion when evaluating superonasal orbital masses, particularly when clinical or imaging features are atypical. Surgeons should be prepared for unexpected intraoperative vascularity and potential hemorrhagic complications. Careful preoperative assessment, intraoperative adaptability, and prompt management of complications are essential to ensure optimal patient outcomes.

## 4. CONCLUSION

This case highlights that traumatic neuroma can mimic a dermoid cyst in the superonasal orbit, particularly in the presence of non-specific clinical and imaging features. Definitive diagnosis relies on histopathological evaluation. Recognition of this entity is important for accurate diagnosis and appropriate management.

## DATA AVAILABILITY STATEMENT

All data related to this study are available from the corresponding author upon reasonable request.

## AUTHORS CONTRIBUTIONS

AK: Conceptualization and supervision of the study; clinical evaluation and management of the patient; acquisition and interpretation of clinical and radiological findings; obtaining informed consent; critical review and revision of the manuscript; final approval of the version to be published. AS: Data collection from medical records, imaging studies, and histopathology reports; literature review; preparation of figures and manuscript drafting; contribution to data interpretation; review and approval of the final manuscript.YS: Data collection and organization; literature review; assistance with manuscript drafting and editing; contribution to data interpretation; review and approval of the final manuscript.All authors contributed to the writing of the manuscript, reviewed the final version, and approved it for publication.

## FUNDING

The author(s) received no financial support for the this article.

## ACKNOWLEDGMENT

The authors would like to thank the patient for providing consent for the publication of this case for medical education purposes.

## DISCLOSURE OF AI USE

The authors used an artificial intelligence (AI)-assisted language tool solely to improve grammar, spelling, language clarity, and readability of the manuscript. The scientific content, clinical data, interpretation of findings, discussion, and conclusions were entirely developed and written by the authors. No AI tool was used to generate, analyze, interpret, or modify patient data or scientific content. The authors reviewed and approved the final manuscript and take full responsibility for its content.

## INFORMED CONSENT

Written informed consent for the publication of this case report and accompanying images was obtained from the patient’s legal guardian (parent).

## COMPETING INTEREST

The authors have no conflicts of interest to declare.

## Figures and Tables

**Figure 3.**
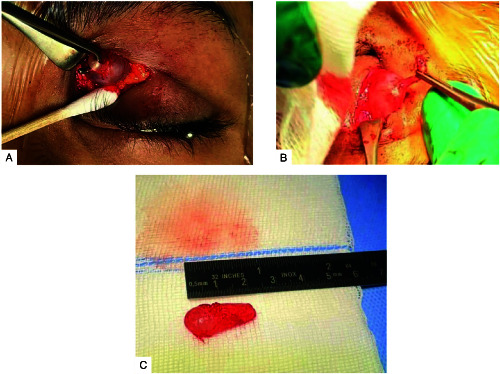
Intraoperative and gross specimen photographs. (A) Exposure of the superonasal orbital mass during surgery. (B) Surgical dissection and mobilization of the lesion. (C) Gross appearance of the excised specimen after complete excision.
